# Salvianolic Acid B Alleviates Atrial Fibrillation–Associated Fibrosis With Modulation of COL1A2 and the PI3K‐AKT Pathway

**DOI:** 10.1155/cdr/5515602

**Published:** 2026-07-08

**Authors:** Pengran Wang, Jianhua Ma, Jianlong Li, Shanshan Li, Yeran Zhu, Ruibin Li, Jidong Zhang

**Affiliations:** ^1^ Department of Cardiology, The Second Hospital of Hebei Medical University, Shijiazhuang, Hebei, China, hebmu.edu.cn; ^2^ Department of Cardiology, Affiliated Hospital of Hebei University, Baoding, Hebei, China, hbu.cn; ^3^ Emergency Department, Affiliated Hospital of Hebei University, Baoding, Hebei, China, hbu.cn

**Keywords:** atrial fibrillation, atrial fibrosis, COL1A2, PI3K-AKT pathway, salvianolic acid B

## Abstract

**Background:**

Atrial fibrillation (AF), the most prevalent cardiac arrhythmia, is strongly associated with atrial fibrosis. Salvianolic acid B (Sal‐B), a bioactive compound extracted from *Salvia miltiorrhiza*, demonstrates cardioprotective properties, though its specific role in AF remains to be elucidated.

**Methods:**

Transcriptome analysis of the GSE115574 dataset was performed to identify *COL1A2* as a differentially expressed gene in patients with AF. Molecular docking simulations were performed to evaluate the binding affinity between Sal‐B and COL1A2. For in vitro experiments, an injury model was established in AC16 cardiomyocytes using Angiotensin II (Ang‐II) treatment, followed by intervention with either Sal‐B (80 *μ*g/mL) or *COL1A2* siRNA. Cellular viability, apoptosis, migration capacity, and fibrosis markers (COL1A1, *α*‐SMA, and collagen III) were subsequently assessed. In vivo studies employed an ACh‐CaCl_2_–induced AF rat model, with cardiac function evaluated through electrocardiography (ECG) and echocardiography. Myocardial tissue damage was examined via hematoxylin–eosin (HE) staining and Masson′s trichrome staining. Western blot analysis was used to determine COL1A2 expression and PI3K‐AKT pathway activity.

**Results:**

*COL1A2* expression was significantly upregulated in AF, and molecular docking demonstrated its strong binding affinity for Sal‐B. Sal‐B treatment rescued Ang‐II–induced cardiomyocyte damage, reduced apoptosis, and suppressed fibrosis marker expression. In rat models, either Sal‐B or si‐*COL1A2* alone shortened the AF duration, improved cardiac function (attenuated the elevated *E*/*E* ^′^ ratio while restoring the ejection fraction), and reduced fibrosis and inflammation. The combined treatment synergistically restored sinus rhythm and normalized collagen deposition, suggesting a modulatory role of Sal‐B in these processes.

**Conclusions:**

Sal‐B alleviates AF in association with COL1A2 modulation and PI3K‐AKT signaling pathway inhibition, which may contribute to reducing atrial fibrosis.

## 1. Introduction

Atrial fibrillation (AF), the most common type of cardiac arrhythmia, significantly increases the risk of stroke, heart failure, and mortality [1]. The pathogenesis of AF is closely associated with myocardial fibrosis, inflammatory responses, and electrical remodeling [2]. Although current pharmacological strategies primarily focus on rhythm control and anticoagulation, these drugs fail to provide a definitive cure and may even carry bleeding risks, representing major clinical limitations [3, 4]. The underlying pathological mechanisms driving AF progression, particularly myocardial fibrosis and structural remodeling, remain unclear.

*COL1A2* encodes the alpha‐2 chain of Type I collagen, one of the most abundant proteins in the extracellular matrix that provides structural integrity to tissues [5]. *COL1A2* expression is strongly correlated with tissue fibrosis [6, 7]. Studies have demonstrated that the transcriptional activation of *COL1A2* promotes myocardial fibrosis following infarction [8]. *COL1A1* and *COL1A2* are key mediators in obesity‐induced cardiac fibrosis [9], and work by Bowers et al. [10] showing that *COL1A2*‐deficient mice develop marked cardiac fibrosis and pathological hypertrophy highlights *COL1A2* as a critical determinant of cardiac structure and injury. Given that atrial fibrosis is central to the initiation and maintenance of AF [11], modulating *COL1A2* expression may offer an effective therapeutic strategy.

Salvianolic acid B (Sal‐B), a major bioactive compound derived from *Salvia miltiorrhiza*, is a promising cardioprotective agent with multifaceted effects [12, 13]. Previous studies have demonstrated its antioxidant, anti‐inflammatory, and antifibrotic properties, suggesting its therapeutic potential in cardiovascular diseases [14, 15]. Moreover, Sal‐B enhances cardiovascular protection by regulating autophagy, mitochondrial function, and apoptosis‐related pathways [16, 17]. Sal‐B attenuates myocardial ischemia‐reperfusion injury [18]. But its effects on atrial fibrosis and the role of *COL1A2* as a molecular target in AF pathophysiology remain unclear.

## 2. Materials and Methods

### 2.1. Screening of Differentially Expressed Genes (DEGs)

Gene expression data from the GSE115574 dataset were obtained from the Gene Expression Omnibus (GEO) database (https://www.ncbi.nlm.nih.gov/geo/). The dataset included atrial tissue samples from patients with persistent AF (*n* = 28) and sinus rhythm (SR) controls (*n* = 31) who underwent mitral valve surgery. Differential expression analysis was performed using the limma package (v3.58.1) with cutoff criteria of |log FC| > 0.5 and *p* value < 0.05 to identify DEGs [19, 20]. Subsequent functional enrichment analysis was conducted using the clusterProfiler R package (v4.10.1) for Kyoto Encyclopedia of Genes and Genomes (KEGG) pathway analysis and gene set enrichment analysis (GSEA).

### 2.2. Drug Target Prediction

Potential targets of Sal‐B were retrieved from the Comparative Toxicogenomics Database (https://ctdbase.org/). The intersection between Sal‐B targets and identified DEGs was determined. The molecular structure file of Sal‐B was obtained from PubChem for use as a ligand molecule, and the crystal structure of COL1A2 was acquired from the Protein Data Bank (https://www.rcsb.org/). Molecular docking simulations were performed using AutoDock Vina (https://github.com/ccsb-scripps/AutoDock-Vina/releases) to evaluate the binding affinity between the ligand and target protein.

### 2.3. Surface Plasmon Resonance (SPR) Assay

The target protein was immobilized on a CM5 chip by standard amine coupling after sequential cleaning with 50 mM NaOH/0.05% SDS and 50 mM NaOH. Flow cells 1 (Fc1, reference) and 2 (Fc2, active) were activated using 1‐ethyl‐3‐(3‐diaminopropyl) carbodiimide hydrochloride (EDC) and N‐hydroxysuccinimide (NHS), and the target protein (30 *μ*g/mL in 10‐mM sodium acetate, pH 4.5) was injected over Fc2 at 5 *μ*L/min for 5 min, followed by ethanolamine blocking. The running buffer was phosphate buffered saline (PBS)/0.05% Tween‐20 (PBST), supplemented with 5% DMSO for small‐molecule binding assays, for which a DMSO solvent calibration curve was generated and the compound was two‐fold serially diluted from 1 *μ*M (including zero) in PBS/5% DMSO. Kinetic measurements were performed at 25°C using a low‐molecular‐weight kinetics program (contact time 60 s, dissociation time 60 s, and regeneration with 10 mM NaOH for 30 s) at a flow rate of 30 *μ*L/min.

### 2.4. Cell Counting Kit‐8 (CCK‐8)

AC16 cells were purchased from Wuhan Procell Life Technology Co. Ltd. These cells have previously been used to evaluate fibrotic markers associated with myocardial fibrosis–related diseases [21, 22]. Cells were seeded into 96‐well plates (FCP962, Beyotime Biotech Inc., China) at a density of 5 × 10^3^ cells per well and incubated at 37°C for 24 h to allow cell attachment. After adherence, the medium was replaced with fresh medium containing different concentrations of Sal‐B (0, 40, 80, 120, and 180 *μ*g/mL; HY‐N1362, MedChemExpress Co. Ltd., China). Following 24, 48, and 72 h of incubation, 10 *μ*L of CCK‐8 solution (A311‐01, Vazyme Biotech Co. Ltd., China) was added to each well and incubated in the dark for 2 h. The absorbance was measured at 450 nm using a microplate reader. To establish a cell injury model, 200 nM Angiotensin II (Ang‐II) (HY‐13948; MedChemExpress Co. Ltd., China) was added to the culture medium for 24 h [23]. The cells were divided into four groups: control (no injury), control + Sal − B (no injury with drug treatment), Ang‐II (injury), and Ang − II + Sal − B (injury with drug treatment).

### 2.5. Wound Healing Assay

AC16 cells were seeded in six‐well plates (FCP060, Beyotime Biotech Inc., China) at a density of 5 × 10^5^ cells/well and cultured for 24 h. A sterile pipette tip was used to create a straight scratch perpendicular to the bottom of each well. Dislodged cells were removed by washing with PBS, and the initial wound was photographed using an inverted microscope. After 48 h of incubation, the same field of view was photographed again. The scratch width was measured and analyzed using ImageJ software v1.54k (National Institutes of Health, Bethesda, Maryland, United States), and the wound healing rate was calculated to determine the cell migration ability.

### 2.6. Flow Cytometry

Cell apoptosis was assessed using the Annexin V‐FITC Apoptosis Detection Kit (556547; Becton, Dickinson and Company, United States). Briefly, cells were harvested by trypsinization without EDTA and resuspended at a concentration of 1 × 10^6^ cells/mL. The cell suspension (100 *μ*L) was incubated with Annexin V‐FITC in 1× Binding Buffer for 15 min in the dark. Propidium iodide (PI) was added, and the cells were incubated for an additional 5 min under the same conditions. The reaction was stopped by adding 400 *μ*L of binding buffer. Samples were immediately analyzed by flow cytometry using 488‐nm excitation to detect Annexin V‐FITC and PI fluorescence signals.

### 2.7. Western Blot

AC16 cells and atrial tissues were lysed in cell and tissue lysis buffers, respectively. Protein concentrations were determined using a BCA protein assay kit (ZJ102L; Epizyme Biotech, China). Equal amounts of protein samples were loaded into each well of the SDS‐PAGE gels for electrophoresis until complete protein separation was achieved. The separated proteins were transferred onto PVDF membranes using a wet transfer system. The membranes were blocked to prevent nonspecific binding and subsequently incubated with primary antibodies at 4°C overnight. Following primary antibody incubation, the membranes were treated with horseradish peroxidase–conjugated secondary antibodies at 25°C for 1 h. All antibodies used are listed in the Supporting Information (Table S1). Protein bands were visualized using ECL chemiluminescence reagents and detected using a chemiluminescence imaging system. The band intensity was quantified using ImageJ software.

### 2.8. Quantitative Real‐Time PCR (qRT‐PCR)

Total RNA was extracted from the samples using TRIzol reagent, and the RNA purity and concentration were assessed by spectrophotometry. Subsequently, RNA was reverse‐transcribed into cDNA using a reverse transcription kit (K1691, Thermo Fisher Scientific, United States). The cDNA was amplified using the primers listed in Table S2 and SYBR Green. The reaction was carried out in a real‐time thermal cycler under standard conditions: initial denaturation at 95°C for 30 s, followed by 40 cycles of 95°C for 5 s and 60°C for 30 s. Relative gene expression was calculated using the 2^−*ΔΔ*Ct^ method, with glyceraldehyde 3‐phosphate dehydrogenase (GAPDH) as the internal control.

### 2.9. Animal Experiments

Male Sprague–Dawley rats (6–8 weeks old, 200–250 g body weight) were purchased from Beijing Vital River Laboratory Animal Technology Co. Ltd. AF was induced by daily tail vein injection of ACh‐CaCl_2_ mixture (ACh 60 *μ*g/mL + CaCl_2_ 10 mg/mL, 0.1 mL/100 g body weight) for 7 consecutive days [24]. An equal volume of normal saline was administered in the control group. Cardiac electrical activity and AF induction were monitored during the experiment, and tissues were collected after the intervention for histological and molecular biological analyses.

Thirty rats were randomly divided into five groups (*n* = 6): control group (normal saline via tail vein injection); AF + si − NC (si‐NC 2 mg/kg via tail vein injection followed by ACh‐CaCl_2_ protocol for AF induction and additional si‐NC (2 mg/kg) on Day 5); AF + Sal − B (Sal‐B 5 mg/kg via tail vein after successful AF induction) [16]; AF + si − COL1A2 (si‐*COL1A2* 2 mg/kg via tail vein injection of 24 h before AF induction, followed by the ACh‐CaCl_2_ protocol and additional si‐*COL1A2* (2 mg/kg) on Day 5); AF + Sal − B + si − COL1A2 (si‐*COL1A2 2* as above plus Sal‐B 5 mg/kg after successful model establishment). si‐*COL1A2* and si‐NC were synthesized by Sangon Biotech Co. Ltd. (China), and their sequences are provided in Table S3. All siRNAs were unmodified and delivered as naked siRNAs without transfection reagents. The knockdown efficiency of COL1A2 in atrial tissues was validated using qPCR.

### 2.10. Electrocardiography (ECG)

ECG was recorded using a small animal ECG monitor (3306B, Shanghai Yuyan Instruments Co. Ltd., China) in rats anesthetized with 2% sevoflurane (Shanghai Hengrui Pharmaceuticals Co. Ltd., China). After shaving and cleaning the chest and limbs with alcohol to reduce skin resistance and improve electrode adhesion, ECG electrodes were placed on the limbs and chest, and tracings were acquired of each rat.

### 2.11. Echocardiography

Rats were anesthetized using the same method. First, an appropriate amount of ultrasound coupling gel was applied to the rat′s chest. The ultrasound probe was then placed on the chest, and clear cardiac images were obtained by adjusting the position and angle. Cardiac structures, including the ventricles, atria, and valves, were observed on the ultrasound images. The dimensions and movements of these structures were measured to calculate the early‐diastolic ratio of inflow velocity to mitral annular velocity (*E*/*E* ^′^), ejection fraction (EF), and left atrial area change fraction (LAACF).

### 2.12. Hematoxylin–Eosin (HE) Staining

Rat atrial tissue was fixed in a 4% paraformaldehyde fixative solution (BL539A, BioSharp Life Sciences, China) for 7 days. The tissue samples were dehydrated through a series of gradient ethanol solutions, cleared in xylene, and then embedded in paraffin and sectioned into 5‐*μ*m slices. After dewaxing and rehydration, the sections were stained with HE (G1120, Beijing Solarbio Science & Technology Co. Ltd., China) to visualize the nuclei and cytoplasm. After staining, the samples were dehydrated again and mounted with a coverslip using a mounting medium to preserve the specimens. Finally, the tissue sections were scanned using a pathological slide scanner (MDS‐MLF05, Medipath Intelligent Technology Co. Ltd., China) to evaluate the tissue morphology and pathological changes.

### 2.13. Masson′s Staining

Masson′s trichrome staining was performed on rat atrial tissue sections. The sections were immersed in Weigert′s iron alum hematoxylin staining solution (AC11556, Shanghai Acmec Biochemical Technology Co. Ltd, China) for 10 min, followed by rinsing with tap water to remove excess dye. The sections were then immersed in acid fuchsin staining solution (P0022; Beyotime Biotech Inc., China) for 2 min and rinsed again with tap water. Subsequently, the sections were immersed in Masson′s staining solution (G1340, Beijing Solarbio Science & Technology Co. Ltd., China) for 10 min and rinsed with tap water. The sections were sequentially dehydrated in a graded ethanol series (70%, 95%, and 100%). The sections were cleared by immersion in xylene for 2 min. Finally, the sections were mounted on glass slides using a neutral resin for preservation.

### 2.14. Enzyme‐Linked Immunosorbent Assay (ELISA)

The rat interleukin (IL)‐6 ELISA Kit (E‐EL‐R0015), rat tumor necrosis factor alpha (TNF‐*α*) ELISA kit (E‐EL‐R2856), Rat IL‐10 ELISA Kit (E‐EL‐R0016), rat cardiac Troponin I (cTnI) ELISA Kit (E‐EL‐R1253), and rat cardiac Troponin T (cTnT) ELISA Kit (E‐EL‐R0151) were all purchased from Elabscience Biotechnology Co. Ltd. The Rat IL‐1*β* ELISA Research Kit (JM‐10482R2) was obtained from Jiangsu Jingmei Biotechnology Co. Ltd.

### 2.15. Statistical Analysis

Statistical analysis was performed using GraphPad Prism Version 9.0 (GraphPad Software, San Diego, California, United States). A two‐tailed Student′s *t* *-*test was employed for comparisons between two groups, whereas a one‐way analysis of variance (ANOVA) followed by Dunnett′s post hoc test was used for multiple‐group comparisons. Data are presented as the mean ± SEM, and a *p* value < 0.05 was considered statistically significant. Western blotting and qRT‐PCR data were normalized to the internal control GAPDH and analyzed using one‐way ANOVA with Bonferroni correction.

## 3. Results

### 3.1. COL1A2 May Serve as a Candidate Target of Sal‐B

In the dataset GSE115574, we identified 115 DEGs (57 upregulated and 58 downregulated) (Figure 1A) with the Top 20 DEGs exhibiting highly consistent and distinct clustering patterns between the two sample groups (Figure 1B). Subsequent KEGG enrichment analysis revealed that these DEGs were primarily enriched in cytoskeleton organization in muscle cells and the PI3K‐AKT signaling pathway (Figure 1C), and GSEA confirmed PI3K‐AKT signaling in AF (NES = 1.75, *p* = 5.69E − 07) (Figure 1D).

Figure 1COL1A2 may serve as a key target of salvianolic acid B (Sal‐B). (A) Volcano plot displaying differentially expressed genes (DEGs) in atrial tissue samples between atrial fibrillation (AF) patients and sinus rhythm controls, with each point representing an individual gene. (B) Heatmap illustrating expression patterns of DEGs across samples, showing the Top 20 DEGs. (C) Dot plot of enriched KEGG pathways, with red boxes highlighting significantly enriched key pathways. (D) Gene set enrichment analysis (GSEA) plot of PI3K‐Akt signaling pathway, displaying running enrichment score. (E) Overlap between DEGs and Sal‐B–related target genes. (F) Three‐dimensional molecular interaction network between COL1A2 and Sal‐B, with binding affinity quantified as −6.78 kcal/mol.

**Figure:**
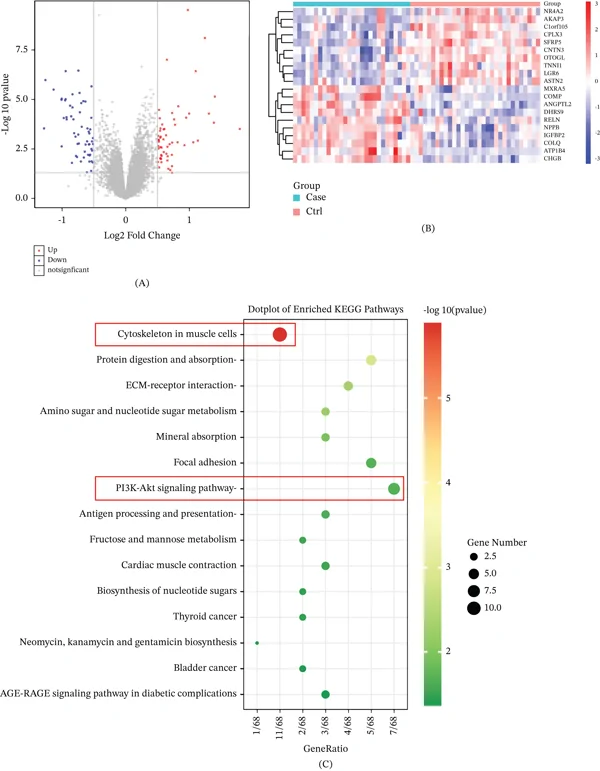

**Figure:**
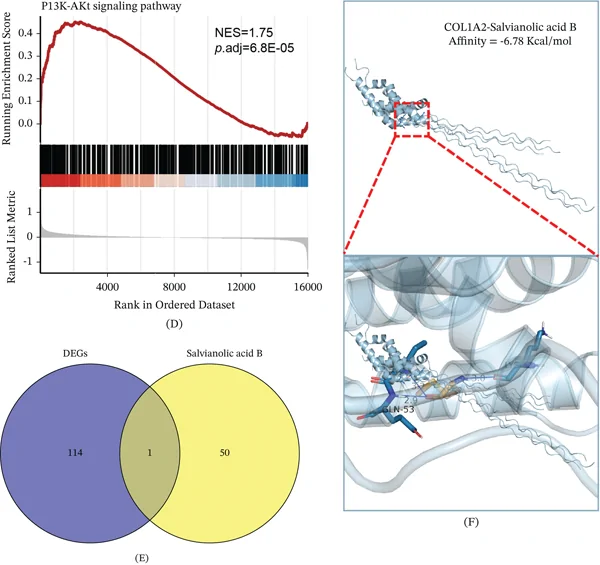

By intersecting Sal‐B targets with the identified DEGs, we identified one overlapping gene, *COL1A2* (|logFC| = 0.73, *p* = 0.02) (Figure 1E), which was significantly upregulated in AF, further supporting its critical role in myocardial cytoskeletal remodeling. Molecular docking of Sal‐B and COL1A2 (Figure 1F) showed strong binding affinity, suggesting COL1A2 as a candidate target of Sal‐B (affinity = −6.78 kcal/mol).

### 3.2. Sal‐B Alleviates Ang‐II–Induced Cardiomyocyte Injury

The 2D and 3D chemical structures of Sal‐B are shown in Figure 2A. SPR was used to further validate the binding of Sal‐B to COL1A2, revealing that Sal‐B directly binds to COL1A2, with an estimated equilibrium dissociation constant (KD) of 2.079 × 10^−7^ M (Figure 2B). AC16 cells were treated with Sal‐B at concentrations of 0, 40, 80, 120, and 180 *μ*g/mL. The CCK‐8 assay results showed that after 24 h of coculture, cell viability decreased by < 10% at all tested concentrations (Figure S1), and viability remained > 90% at concentrations < 80 *μ*g/mL after 48 and 72 h of coculture, whereas higher doses (> 80 *μ*g/mL) led to > 10% loss of viability. Accordingly, we selected 80 *μ*g/mL Sal‐B with a 48‐h incubation period for subsequent experiments (Figure 2C). Next, we established a cardiomyocyte injury model by treating AC16 cells with 1 *μ*M Ang‐II for 24 h. Compared with the control group, the Ang‐II group exhibited markedly decreased cell viability, whereas the Ang − II + Sal − B group showed restored cell viability, indicating that Sal‐B attenuates Ang‐II–induced cardiomyocyte injury (Figure 2D).

Figure 2Sal‐B can alleviate Ang‐II–induced cardiomyocyte injury. (A) Chemical structures of Sal‐B, including its 2D and 3D representations. The molecular formula is provided, along with its relative molecular mass. (B) Surface plasmon resonance assay showing binding of Sal‐B to COL1A2. (C) Cell viability assessed using the CCK‐8 assay after treating cells with various concentrations of Sal‐B (0, 40, 80, 120, 180 *μ*g/mL) for 48 and 72 h. (D) Cell viability measured by the CCK‐8 assay in different treatment groups. All data are given as the mean ± SD (*n* = 6). Statistical significance is indicated by “ns” (not significant) and  ^∗^ ( ^∗∗^ *p* < 0.01,  ^∗∗∗^ *p* < 0.001,  ^∗∗∗∗^ *p* < 0.0001).

**Figure:**
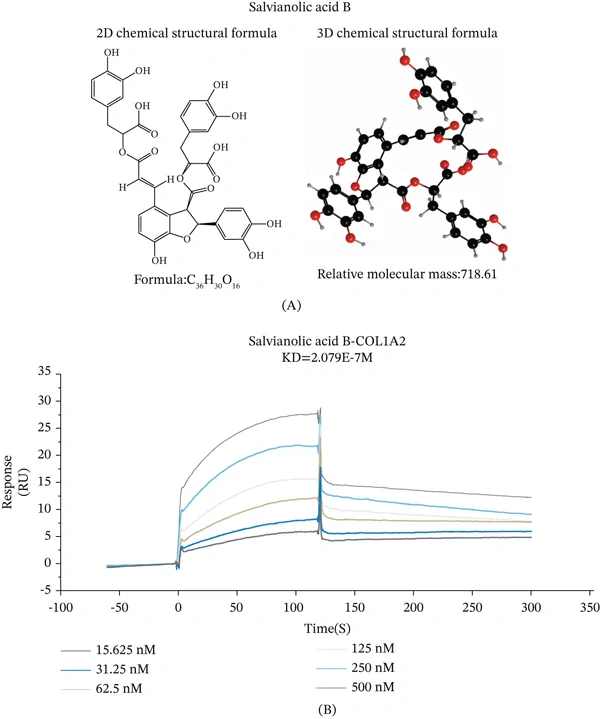

**Figure:**
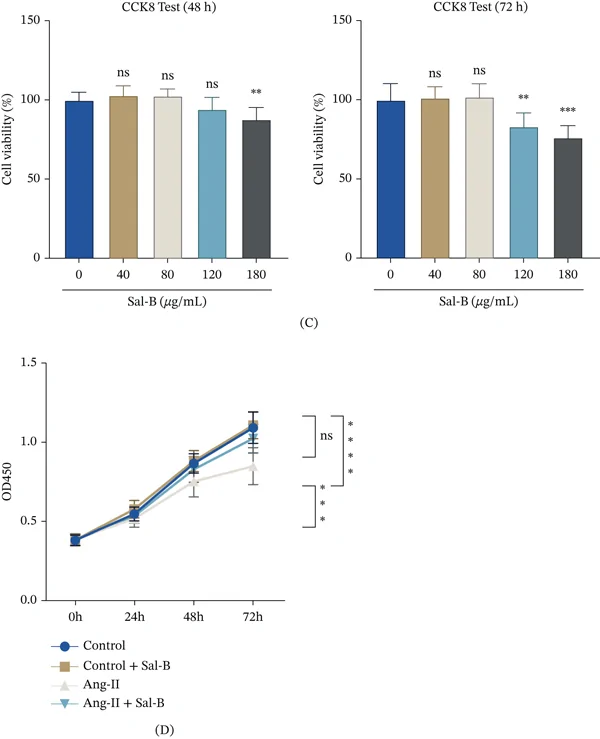

### 3.3. Sal‐B Inhibits Cardiomyocyte Apoptosis by Targeting COL1A2

**Figure 3:**
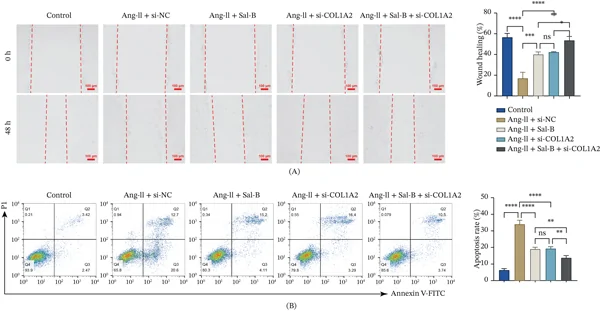
Sal‐B inhibits cardiomyocyte apoptosis by targeting COL1A2. (A) The figure shows cell healing conditions at 0 and 48 h in different treatment groups. (B) Flow cytometry measured apoptosis rates in different treatment groups. All data are given as the mean ± SD (*n* = 6). Statistical significance is indicated by “ns” (not significant) and  ^∗^ ( ^∗^ *p* < 0.05,  ^∗∗^ *p* < 0.01,  ^∗∗∗∗^ *p* < 0.0001).

Next, we performed *COL1A2* knockdown experiments in AC16 cells and evaluated the knockdown efficiency using qRT‐PCR, ultimately selecting the si‐*COL1A2-2* sequence for subsequent experiments (Figure S2). The wound healing assay revealed that the Ang‐II group exhibited a significantly reduced healing rate in AC16 cells compared with the control group. However, Sal‐B intervention or si‐*COL1A2* treatment after Ang‐II exposure restored the healing rate, with the combined Sal‐B and *COL1A2* silencing showing the highest cell migration rates (Figure 3A). In the flow cytometry analysis of apoptosis, Ang‐II markedly increased apoptosis, whereas Sal‐B or si‐*COL1A2* alone effectively reduced apoptosis (Figure 3B).

### 3.4. Sal‐B Suppresses the PI3K‐AKT Signaling Pathway by Targeting COL1A2

**Figure 4:**
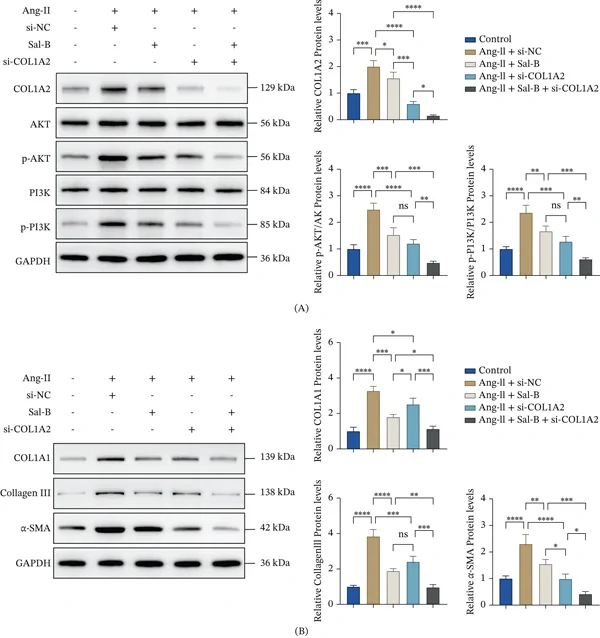
Sal‐B inhibits PI3K‐AKT signaling pathway by targeting COL1A2. (A) Western blot analysis of COL1A2 and PI3K‐AKT signaling pathway‐related protein expression. (B) Western blot analysis of fibrosis‐related protein expression. All data are given as the mean ± SD (*n* = 6). Statistical significance is indicated by “ns” (not significant) and  ^∗^ ( ^∗^ *p* < 0.05,  ^∗∗^ *p* < 0.01,  ^∗∗∗^ *p* < 0.001,  ^∗∗∗∗^ *p* < 0.0001).

Our findings extend prior evidence implicating the PI3K‐AKT signaling pathway in myocardial fibrosis and AF. Previous studies demonstrated the involvement of the PI3K‐AKT pathway in myocardial fibrosis and AF [25, 26]. Our key gene, *COL1A2*, has been implicated in the regulation of this pathway, prompting us to examine its activation status via western blotting. Validation of the knockdown efficiency of si‐*COL1A2* is shown in Figure S3. Compared with the Ang − II + si − NC group, both Sal‐B treatment and si‐COL1A2 significantly reduced the expression levels of p‐PI3K and p‐AKT in AC16 cells. The Ang − II + Sal − B + si − *C* *O* *L*1*A*2 group exhibited the lowest expression of these phosphorylated proteins (Figure 4A). We further assessed the expression of fibrosis‐related proteins in cardiomyocytes. Relative to the control group, the Ang‐II group showed significant upregulation of COL1A1, *α*‐SMA, and collagen III protein levels. In contrast, the Ang − II + Sal − B group displayed a marked downregulation of these proteins. The Ang − II + si − *C* *O* *L*1*A*2 group not only suppressed COL1A1 but also significantly reduced *α*‐SMA and collagen III levels. Combined treatment with Sal‐B and si‐COL1A2 (Ang − II + Sal − B + si − *C* *O* *L*1*A*2) further minimized the expression of COL1A1, *α*‐SMA, and collagen III (Figure 4B).

### 3.5. Sal‐B Alleviates AF in Rats by Inhibiting COL1A2 Expression

**Figure 5:**
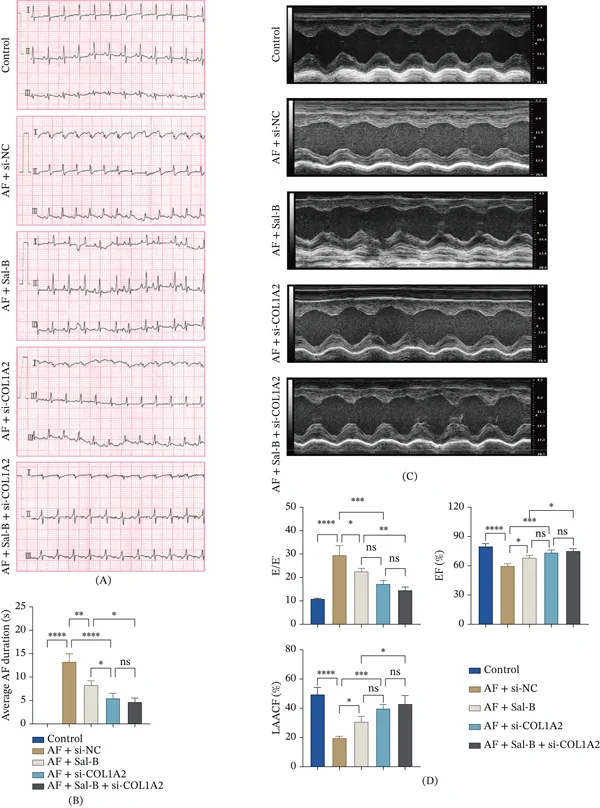
Sal‐B alleviates atrial fibrillation (AF) in rats by inhibiting COL1A2 expression. (A) Representative electrocardiography from each group of rats. (B) Recording and statistical analysis of atrial fibrillation duration in rats over 30 min. (C) Representative echocardiograms from each group of rats. (D) Calculation of cardiac structural and functional parameters including early‐diastolic ratio of inflow velocity to mitral annular velocity (*E*/*E* ^′^), ejection fraction (EF), and left atrial area change fraction (LAACF). All data are given as the mean ± SD (*n* = 6). Statistical significance is indicated by “ns” (not significant) and  ^∗^ ( ^∗^ *p* < 0.05,  ^∗∗^ *p* < 0.01,  ^∗∗∗^ *p* < 0.001,  ^∗∗∗∗^ *p* < 0.0001).

ECGs were recorded to characterize the atrial and ventricular electrical phenotypes of rats. Representative traces are shown in Figure 5A. Control rats exhibited uniform P‐waves preceding each QRS complex, stable R‐R intervals, and isoelectric baselines between cycles, confirming a normal SR. The AF + si − NC group displayed intermittent AF, characterized by the complete absence of discernible P‐waves replaced by irregular f‐waves. AF + Sal − B monotherapy attenuated but did not eliminate AF. Low‐amplitude f‐waves persisted, but organized electrical activity reappeared intermittently, generating quasi‐P‐waves. The AF + si − *C* *O* *L*1*A*2 group showed significantly suppressed but incompletely restored SR, with observable low‐amplitude, morphologically inconsistent P‐waves, and residual very‐low‐amplitude fibrillation waves. AF + Sal − B + si − *C* *O* *L*1*A*2 combination therapy restored near‐normal SR, with the reappearance of sharp and uniform P‐waves and the near disappearance of f‐waves. Quantitative analysis of AF duration during the 30‐min recording period showed that control rats maintained SR, whereas AF + si − NC rats exhibited AF durations exceeding 12 s. Sal‐B monotherapy reduced the AF duration, and *COL1A2* knockdown produced nearly identical reductions, with no significant difference between the two monotherapies. The combination of Sal‐B and si‐*COL1A2* further shortened the duration to approximately 5 s (Figure 5B). The representative ECGs are shown in Figure 5C. Control rats demonstrated normal left ventricular filling (low *E*/*E* ^′^), preserved EF, and maintained LAACF. Conversely, AF + si − NC animals exhibited characteristic AF‐related dysfunction: significantly elevated *E*/*E* ^′^, with markedly reduced LAACF and EF. Both Sal‐B treatment and COL1A2 knockdown suppressed *E*/*E* ^′^ elevation, while restoring LAACF and EF. No significant differences were observed between the combination therapy (AF + Sal − B + si − COL1A2) and gene therapy (AF + si − *C* *O* *L*1*A*2) groups (Figure 5D). These results demonstrate that Sal‐B alleviates rat AF through COL1A2 regulation.

### 3.6. Sal‐B Alleviates Inflammation and Fibrosis in Rat Atrial Tissue by Inhibiting COL1A2 Expression

The primary function of COL1A2 is to serve as a tissue scaffold that provides structural tension. It is closely associated with cell growth, differentiation, tissue repair, inflammatory response, sclerosis, and fibrosis. Based on these functions, COL1A2 may be linked to atrial fibrosis [10]. Figure 6A shows representative HE‐stained images, and Figure 6B shows representative Masson′s‐stained images of the left atrial tissue. In the control group, HE staining revealed uniformly arranged cardiomyocytes with minimal gaps, whereas Masson′s trichrome staining showed only sparse collagen fibers. In the AF + si − NC model, HE staining revealed widespread myocyte disarray and significant interstitial edema, whereas Masson′s‐stained sections exhibited dense, irregular collagen deposition. Sal‐B treatment reversed these changes; compared with AF + si − NC, myocyte alignment improved, interstitial edema decreased, and collagen accumulation was markedly reduced. COL1A2 knockdown alone (AF + si − *C* *O* *L*1*A*2) improved myocyte alignment and reduced fibrosis, and combined combination therapy (AF + Sal − B + si − COL1A2) provided robust protection; the tissue structure closely resembled that of the control group, with minimal inflammatory infiltration and near‐complete elimination of collagen deposition (Figure S4).

Figure 6Sal‐B alleviates inflammation and fibrosis in rat atrial tissue by inhibiting COL1A2 expression. (A) Hematoxylin–eosin staining of rat atrial tissue. (B) Masson staining of rat atrial tissue. (C) ELISA detection of cytokines (IL‐6, IL‐1*β*, TNF‐*α*, and IL‐10) and myocardial injury markers (cTnI and cTnT) in atrial tissue. All data are given as the mean ± SD (*n* = 6). Statistical significance is indicated by “ns” (not significant) and  ^∗^ ( ^∗^ *p* < 0.05,  ^∗∗^ *p* < 0.01,  ^∗∗∗^ *p* < 0.001,  ^∗∗∗∗^ *p* < 0.0001).

**Figure:**
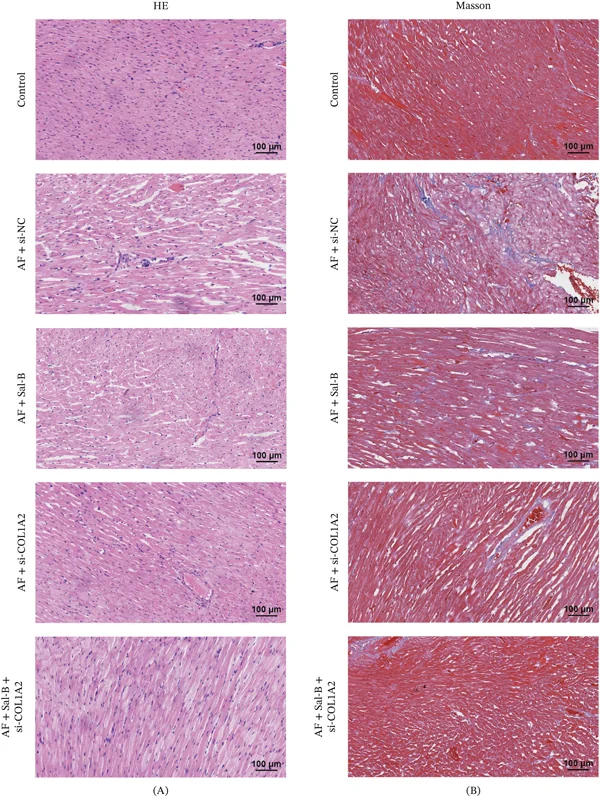

**Figure:**
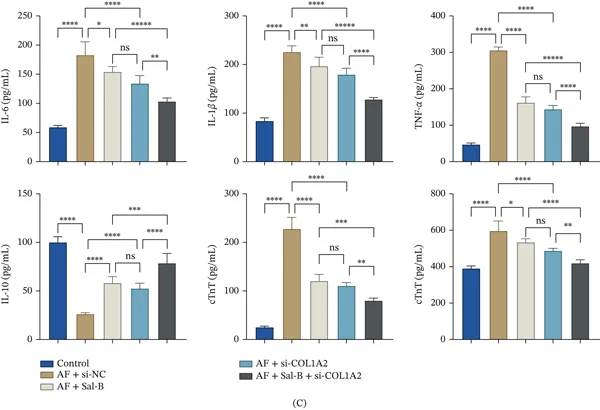

The ELISA results for atrial tissue inflammatory and injury markers are shown in Figure 6C. Compared with the control group, the AF + si − NC group exhibited significant upregulation of IL‐6, IL‐1*β*, and TNF‐*α*, elevated cTnI and cTnT, and marked downregulation of IL‐10, indicating pronounced atrial injury and inflammation. Sal‐B monotherapy (AF + Sal − B) reduced proinflammatory cytokines and injury markers, while partially restoring IL‐10 levels. *COL1A2* knockdown alone (AF + si − *C* *O* *L*1*A*2) similarly decreased IL‐6, IL‐1*β*, TNF‐*α*, cTnI, and cTnT, though IL‐10 remained lower than control levels. The combination therapy (AF + Sal − B + si − *C* *O* *L*1*A*2) demonstrated optimal anti‐inflammatory and cardioprotective effects, with IL‐6, IL‐1*β*, TNF‐*α*, cTnI, and cTnT all reduced to control levels and IL‐10 restored to near‐control values.

### 3.7. Sal‐B Alleviates Atrial Fibrosis in AF by Targeting COL1A2 to Inhibit the PI3K‐AKT Signaling Pathway

**Figure 7:**
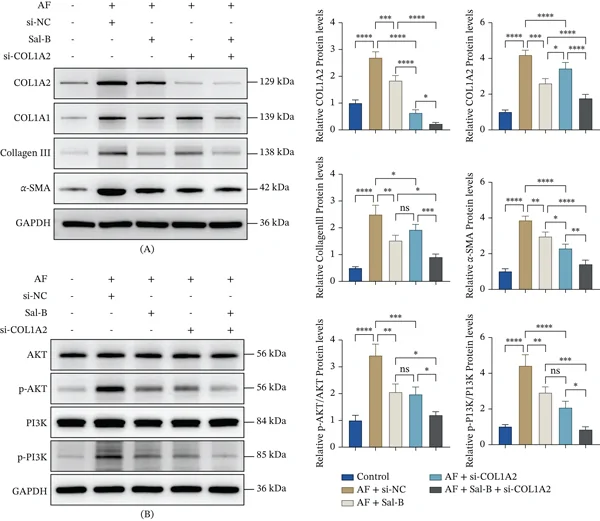
Sal‐B attenuates AF‐associated fibrosis by targeting COL1A2 to inhibit the PI3K‐AKT signaling pathway. (A) Western blot analysis of COL1A2 and fibrosis‐related protein expression. (B) Western blot analysis of PI3K‐AKT signaling pathway‐related protein expression. All data are given as the mean ± SD (*n* = 6). Statistical significance is indicated by “ns” (not significant) and  ^∗^ ( ^∗^ *p* < 0.05,  ^∗∗^ *p* < 0.01,  ^∗∗∗^ *p* < 0.001,  ^∗∗∗∗^ *p* < 0.0001).

Using Western blot analysis, we examined the expression levels of fibrosis‐related proteins (COL1A2, COL1A1, collagen III, and *α*‐SMA) (Figure 7A) and PI3K‐AKT pathway‐related proteins (Figure 7B) in left atrial tissues. Sal‐B significantly inhibited COL1A2 expression, markedly suppressed the upregulation of all fibrosis markers, and reduced p‐AKT and p‐PI3K levels to below those in the AF + si − NC group. COL1A2 knockdown alone (AF + si − COL1A2) similarly reduced COL1A2, COL1A1, collagen III, and *α*‐SMA, with comparable decreases in p‐AKT and p‐PI3K; notably, the reductions in COL1A1 and *α*‐SMA were slightly more pronounced than with Sal‐B monotherapy. The AF + Sal − B + si − *C* *O* *L*1*A*2 group exhibited the strongest inhibitory effects: Fibrosis‐related proteins (COL1A1, collagen III, and *α*‐SMA) were restored to near‐control levels, whereas p‐AKT and p‐PI3K were also significantly suppressed.

## 4. Discussion

In this study, we demonstrated that Sal‐B alleviated atrial fibrosis and reduced AF susceptibility in association with downregulating COL1A2 and inhibiting the PI3K‐AKT signaling pathway in both cellular and rat models. These findings suggest that COL1A2 and the PI3K‐AKT pathway may be important mediators of the protective effects of Sal‐B. Bioinformatics analysis and molecular docking identified *COL1A2* as a Sal‐B–interacting, AF‐related DEG, which was further validated by SPR experiments. Experimental validation revealed that Sal‐B suppresses *COL1A2* expression, dampens PI3K‐AKT pathway activation, and mitigates fibrotic changes in both cellular and animal models of AF. These findings suggest that Sal‐B, through its effects on *COL1A2*‐mediated fibrosis, represents a promising therapeutic agent for AF.

In clinical practice, AF is frequently complicated stroke and heart failure, highlighting the need for effective treatment strategies [27]. Recent advances have highlighted Sal‐B as a cardioprotective agent, including evidence that it inhibits cardiomyocyte apoptosis via modulation of the TRIM8/GPX1 signaling pathway [28]. Sal‐B has also been shown to regulate macrophage polarization in ischemia/reperfusion–injured hearts by suppressing mTORC1‐induced glycolysis [29]. In rat models, Sal‐B effectively reduces myocardial infarct size, improves cardiac function, and lowers the levels of injury markers [30]. Sal‐B also exhibits notable anti‐inflammatory and antioxidant properties, which are particularly valuable in the treatment of cardiovascular diseases. Prior studies show that Sal‐B limits inflammatory factors release from vascular endothelial cells [31] and mitigates oxidative stress damage via Nrf2/ARE signaling pathway [13]. Consistent with these reports, our study further confirms that Sal‐B suppresses the secretion of inflammatory factors (IL‐6, IL‐1*β*, and TNF‐*α*) in myocardial tissue. In myocardial fibrosis, Sal‐B inhibits the proliferation, migration, and collagen secretion of cardiac fibroblasts by upregulating Smad7 and suppressing the TGF‐*β*1 signaling pathway [32]. Our results provide histopathological evidence that Sal‐B effectively attenuates ACh‐CaCl2–induced myocardial fibrosis while reducing the levels of the myocardial injury markers cTnI and cTnT.

Atrial fibrosis constitutes a critical pathological basis for the initiation and progression of AF. Fibrotic remodeling leads to abnormal collagen deposition in the atrial tissue, disrupting normal cardiomyocyte alignment and creating heterogeneous electrical conduction, thereby increasing AF susceptibility [11]. Our study identified that *COL1A2* plays a pivotal role in AF‐associated fibrosis, consistent with its established role in extracellular matrix deposition and fibroblast activation. Previous studies show that *COL1A2* upregulation promotes collagen accumulation, resulting in tissue stiffening and electrical dysfunction [33], and its recognition as a biomarker of heart failure and myocardial injury [34], are in line with our results, which link *COL1A2* expression to structural remodeling in AF.

The role of the PI3K‐AKT pathway in fibrosis is a well‐established regulator of fibroblast proliferation and collagen synthesis through activation of downstream molecules, including mTOR, thereby exacerbating cardiac fibrosis [35]. Activated AKT also enhances cardiomyocyte contractility and anti‐apoptotic capacity, exerting protective effects under pathological conditions, such as myocardial ischemia/reperfusion injury and infarction [36]. The PI3K‐AKT pathway also participates in inflammatory regulation, where Akt can activate NF‐*κ*B signaling to promote inflammatory factor release, aggravating cardiac inflammation and fibrosis [37]. In our AF rat model, PI3K‐AKT activation was accompanied by inflammatory infiltration and aggravated fibrosis, whereas Sal‐B treatment reduced COL1A2 expression, suppressed PI3K‐AKT signaling, and thereby attenuated atrial fibrosis in rats with AF.

Despite these promising findings, this study has several limitations. Our in vitro experiments relied on AC16 cardiomyocytes rather than primary atrial fibroblasts, and future studies should validate these findings in more disease‐relevant cell types. In addition, although Sal‐B treatment and COL1A2 knockdown reduce PI3K‐AKT activation and fibrosis‐related protein expression, the additive effects observed with combination therapy suggest that Sal‐B may act through multiple mechanisms, and our data do not establish that Sal‐B acts exclusively through COL1A2 to regulate this pathway. Additional mechanistic validation, such as rescue experiments (e.g., *COL1A2* overexpression following Sal‐B treatment) and exploration of other potential pathways, will be necessary to clarify the precise molecular hierarchy and potential redundant mechanisms. Finally, given the crosstalk between the PI3K‐AKT pathway and other signaling cascades, further research is required to elucidate broader signaling networks engaged in AF‐related mechanisms.

## Author Contributions

Pengran Wang and Jidong Zhang participated in the study design; Pengran Wang, Jianhua Ma, and Jianlong Li performed the experiments and data processing; Shanshan Li, Ruibin Li, and Yeran Zhu primarily completed the data analysis and data visualization; Pengran Wang drafted the initial manuscript; and Pengran Wang and Jidong Zhang proofread and reviewed the manuscript.

## Funding

This study was supported by the Medical Science Research Project of Hebei (No. GZ2023011).

## Ethics Statement

The study protocol was reviewed and approved by the Experimental Animal Welfare Ethics Committee of Hebei University (HBU2024R055).

## Conflicts of Interest

The authors declare no conflicts of interest.

## Supporting information


**Supporting Information** Additional supporting information can be found online in the Supporting Information section. Table S1: Antibodies information for Western blot. Table S2: Primers used for quantitative real‐time PCR. Table S3: Sequences of si‐COL1A2 and si‐NC. Figure S1: After treating AC16 cells with different concentrations of Sal‐B for 0 and 24 h, the cell viability was detected using CCK‐8. Figure S2: Validation of COL1A2 knockdown efficiency in AC16 cells by qRT‐PCR. Figure S3: Validation of COL1A2 knockdown efficiency in animal model tissues by qRT‐PCR. Figure S4: Quantitative analysis of collagen volume fraction using ImageJ.

## Data Availability

The datasets used and analyzed in the current study are available from the corresponding author upon reasonable request.
