# Supplementary material for: Salvianolic Acid B Alleviates Atrial Fibrillation–Associated Fibrosis With Modulation of COL1A2 and the PI3K‐AKT Pathway
Source: Cardiovasc Ther. 2026 Jul 8;2026:5515602. doi: 10.1155/cdr/5515602 (PMC13598933; doi:10.1155/cdr/5515602)
Supplement: Supplementary file 1 [file CDR-2026-5515602-s001.docx]

**Table S1.** **Antibodies information for Western blot**

| Antibody | Dilution ratio | Product number | Manufacturer |
| --- | --- | --- | --- |
| anti-COL1A2 | 1:2000 | HA751142 | HUABIO |
| anti-COL1A1 | 1:1000 | HA722517 | HUABIO |
| anti-Collagen III | 1:1000 | 22734-1-AP | proteintech |
| anti-α-SMA | 1:1000 | AF1032 | Affinity |
| anti-AKT | 1:1000 | AF6261 | Affinity |
| anti-p-AKT | 1:1000 | AF0016 | Affinity |
| anti-PI3K | 1:1000 | AF6241 | Affinity |
| anti-p-PI3K | 1:1000 | AF3241 | Affinity |
| GAPDH | 1:10000 | ab8245 | Abcam |
| Goat Anti-Rabbit IgG H&L | 1:5000 | ab205718 | Abcam |
| Goat Anti-Mouse IgG H&L | 1:5000 | ab205719 | Abcam |

**Table S2. Primers used for quantitative real‑time PCR**

| Species | Name | Sequence (5'-3')-F | Sequence (5'-3')-R |
| --- | --- | --- | --- |
| Human | COL1A2 | GAGGAGAGCCTGGCAACATT | AGGACCAGGGAGACCAAACT |
|  | GAPDH | GTCAAGGCTGAGAACGGGAA | AAATGAGCCCCAGCCTTCTC |
| Rat | COL1A2 | GAAATGGCAACTCAGCTCGC | CTGAGAAGCACGGTTGGCTA |
|  | GAPDH | TGTGAACGGATTTGGCCGTA | GATGGTGATGGGTTTCCCGT |

**Table S3. Sequences of si‑COL1A2 and si‑NC**

| Species | Name | SS Sequence | AS Sequence |
| --- | --- | --- | --- |
| Human | si-COL1A2-1 | GAAUGUUGAUGGUGCUAGAAG | UCUAGCACCAUCAACAUUCUU |
|  | si-COL1A2-2 | GUUUGAAUAUAAUGUAGAAGG | UUCUACAUUAUAUUCAAACUG |
|  | si-COL1A2-3 | GGUGCUAAUUAAUAGUAUUUC | AAUACUAUUAAUUAGCACCUU |
|  | si-NC | UUCUCCGAACGUGUCACGUTT | ACGUGACACGUUCGGAGAATT |
| Rat | si-COL1A2-1 | GGUUAGGAGAGACCAUCAAUG | UUGAUGGUCUCUCCUAACCAG |
|  | si-COL1A2-2 | CGGUGGUACUAACCAAGAAUU | UUCUUGGUUAGUACCACCGAU |
|  | si-COL1A2-3 | GGACAAGACAAUCAUUGAAUA | UUCAAUGAUUGUCUUGUCCCA |
|  | si-NC | UUCUCCGAACGUGUCACGUTT | ACGUGACACGUUCGGAGAATT |

**Figure S1. After treating AC16 cells with different concentrations of Sal-B for 0 and 24 h, the cell viability was detected using CCK-8.**

**Figure S2. Validation of COL1A2 knockdown efficiency in AC16 cells by qRT-PCR**

**Figure S3. Validation of COL1A2 knockdown efficiency in animal model tissues by qRT-PCR**

**Figure S4. Quantitative analysis of collagen volume fraction using ImageJ.**
